# Impact of maternal hypothyroidism during pregnancy on neonatal corpus callosum development: a transcranial ultrasound analysis

**DOI:** 10.3389/fnins.2026.1739196

**Published:** 2026-03-19

**Authors:** Qian-Feng Ma, Li-Yuan Ma, Guang-Fei Yang, Hui Zhang, Hai-Bo Ma, Li-Tao Ruan

**Affiliations:** 1 Ultrasound Imaging Department, The First Affiliated Hospital of Xi’an Jiaotong University, Xi’an, Shaanxi, China; 2 Department of Ultrasonography, General Hospital of Ningxia Medical University, Yinchuan, China; 3 Department of Ultrasonography, Graduate School of Ningxia Medical University, Yinchuan, China

**Keywords:** cerebral reactivity, corpus callosum, maternal hypothyroidism during pregnancy, neonate, transcranial ultrasound

## Abstract

**Objective:**

This study aimed to examine the association between maternal hypothyroidism during pregnancy and corpus callosum development in neonates.

**Methods:**

The hypothyroidism group included 64 neonates born to women diagnosed with hypothyroidism during pregnancy, while the control group comprised 101 neonates born to women without a history of hypothyroidism. Transcranial ultrasound (TCUS), together with medical imaging analysis software, was used to evaluate corpus callosum development, cerebral reactivity, and early neurodevelopmental patterns during the neonatal period.

**Results:**

Assessment of corpus callosum development across early postnatal stages showed no statistically significant differences in growth rates between preterm and full-term neonates during the first 0 to 2 weeks of life (*p* > 0.05). In contrast, between 2 and 6 weeks postnatally, significant differences in weekly corpus callosum growth rates were observed (all *p* < 0.001; Cohen’s *d* = 0.97–1.12). The mean corpus callosum growth rate from birth to 6 weeks was 0.56 mm/week (0.08 mm/day) in preterm neonates and 1.19 mm/week (0.17 mm/day) in full-term neonates. Evaluation of cerebral reactivity demonstrated lower regional cerebral tissue oxygen saturation (rSO₂), prolonged latency periods, and reduced peak rSO₂ response values in neonates in the hypothyroidism group compared with controls, with all differences reaching statistical significance (*p* < 0.01). Findings were consistent with delayed cerebral maturation and measurable neurodevelopmental differences.

**Conclusion:**

Maternal hypothyroidism during pregnancy is associated with atypical corpus callosum development and altered cerebral reactivity in neonates. These findings should be interpreted cautiously, as the observational design precludes causal inference and residual confounding cannot be excluded.

## 1 Introduction

The corpus callosum, the brain’s largest white matter structure, consists of a dense bundle of nerve fibers located beneath the cerebral cortex that enables communication between the left and right cerebral hemispheres. Its appropriate development is essential for cognitive function and motor coordination during the neonatal period. Structural anomalies of the corpus callosum, including hypoplasia or agenesis, have been associated with epilepsy, intellectual disability, and motor coordination disorders in neonates (Lei and Wen, 2021). Early identification, diagnosis, and clinical management of atypical corpus callosum development are therefore critical for promoting optimal neurodevelopmental outcomes.

Hypothyroidism is a systemic hypometabolic condition marked by insufficient production or physiological activity of thyroid hormones, resulting from a range of potential etiologies. During pregnancy, the demand for thyroid hormones increases to support maternal and fetal metabolic needs. When this increased demand is unmet—due to impaired synthesis, secretion, or regulatory dysfunction—maternal hypothyroidism may occur. Inadequate maternal thyroid hormone levels during gestation can negatively affect fetal neurodevelopment and have been associated with congenital cognitive impairments (Du and Yang, 2024). Accordingly, regular assessment of thyroid function is a key component of prenatal care intended to safeguard fetal and neonatal development.

The present study investigates the relationship between maternal hypothyroidism during pregnancy and corpus callosum development in neonates, with the aim of contributing to early identification of neurodevelopmental risk factors.

## 2 Data and method

### 2.1 Participants and grouping

The hypothyroidism group included 64 neonates born to women diagnosed with hypothyroidism during pregnancy, all of whom were admitted to the Neonatology Department of the study hospital between June 2016 and December 2018. Maternal hypothyroidism was diagnosed according to the 2017 guidelines of the American Thyroid Association for the diagnosis and management of thyroid disease during pregnancy and postpartum (Alexander et al., 2017). Diagnostic criteria comprised overt hypothyroidism—defined as thyroid-stimulating hormone (TSH) levels >2.5 mIU/L in the first trimester or >3.0 mIU/L in the second or third trimester, with free thyroxine (FT4) levels below the lower reference limit—and subclinical hypothyroidism, defined as TSH levels above trimester-specific cutoffs with FT4 values remaining within the normal range.

Among the 64 neonates, 38 were male and 26 were female. Distribution by gestational age at birth was as follows: 26 neonates were born at <34 weeks, 23 between 34 and 37 weeks, and 15 at ≥37 weeks. Regarding maternal thyroid status, 28 women (43.8%) were diagnosed with overt hypothyroidism and 36 (56.2%) with subclinical hypothyroidism. The timing of diagnosis was as follows: first trimester (*n* = 21, 32.8%), second trimester (*n* = 30, 46.9%), and third trimester (*n* = 13, 20.3%). A total of 41 women (64.1%) received levothyroxine replacement therapy, initiated at a median dose of 50 μg/day and adjusted as needed to maintain TSH within trimester-specific reference ranges. The remaining 23 women (35.9%) did not receive treatment, primarily due to late diagnosis or refusal of therapy.

The control group comprised 101 neonates born to women without a diagnosis of hypothyroidism during pregnancy. Exclusion criteria for this group included the presence of severe systemic conditions known to affect neurodevelopment independently, such as severe sepsis, hypoxic–ischemic encephalopathy, or congenital malformations. Neonates in the control group were admitted primarily for conditions including pneumonia, hyperbilirubinemia, or respiratory distress. Distribution by gestational age was as follows: 19 neonates were born at <34 weeks, 43 between 34 and 37 weeks, and 39 at ≥37 weeks. Approximate birth weights by gestational age were: 2400 ± 200 g for neonates born at <34 weeks, 3,000 ± 250 g for those born between 34 and 37 weeks, and 3,400 ± 100 g for those born at ≥37 weeks.

### 2.2 Instruments and methods

#### 2.2.1 Ultrasound equipment

A GE LOGIQe bedside color Doppler ultrasound system, equipped with a convex array probe operating at a central frequency of 7.5 MHz, was used for imaging.

#### 2.2.2 Ultrasound examination protocol

Transcranial ultrasound (TCUS) was performed on all neonates within the first 24 h after birth and then repeated weekly until 6 weeks of age. Examinations were conducted while neonates were in a quiet state and placed supine. The ultrasound probe was positioned over the anterior fontanelle to obtain continuous scans in both coronal and sagittal planes, producing standardized cranial sonographic images.

In the coronal plane, the frontal lobe section allowed visualization of the frontal lobes and interhemispheric fissure. The anterior horn section included the head of the caudate nucleus, corpus callosum, anterior horns of the lateral ventricles, cavum septi pellucidi, and lateral fissure. The third ventricle section displayed the thalamus, caudate nucleus, third ventricle, pons, and temporal lobes. The central–posterior horn section revealed bilateral choroid plexuses and lateral ventricles, while the parietal section showed the parietal lobes and hemispheric fissures.

In the sagittal plane, the mid-sagittal section demonstrated the corpus callosum, cingulate gyrus, cavum septi pellucidi, third ventricle, cerebellar hemispheres, fourth ventricle, pons, and midbrain. Additional sagittal views captured the anterior horn of the lateral ventricle (including the caudate nucleus, thalamus, temporal lobes, and caudate sulcus), as well as the central–posterior horn region, showing the choroid plexus, lateral ventricle, and surrounding lobes. The insular section provided visualization of the temporal lobes.

#### 2.2.3 Ultrasound measurements

The morphology of the corpus callosum was evaluated to determine whether it was present or absent. Corpus callosum length, defined as the distance from the genu to the splenium, was measured in the mid-sagittal plane. Weekly measurements were averaged for each neonate through 6 weeks of life. All measurements were performed independently by two experienced radiologists who were blinded to group allocation. Inter-rater reliability was assessed using the intraclass correlation coefficient, yielding a value of 0.92 (95% CI: 0.87–0.95), indicating excellent agreement. Any discrepancies were resolved by consensus following discussion with a third senior radiologist.

#### 2.2.4 Cerebral reactivity assessment

Cerebral reactivity was evaluated using baseline regional cerebral tissue oxygen saturation (rSO₂) and its changes in response to auditory stimulation. Measurements were conducted with the TSNIR-3 near-infrared tissue oxygenation monitor, developed by the Human Motion Information Detection Laboratory at Tsinghua University. Near-infrared spectroscopy was used to measure baseline rSO₂, followed by auditory stimulation during which rSO₂ was continuously recorded to assess latency (time to initial response) and the maximum change in saturation (de Almeida et al., 2023). The auditory stimulus consisted of an 8-min excerpt from Mozart’s *Sonata for Two Pianos in D Major*, K. 448, selected based on previous studies demonstrating its neuroregulatory effects in infants (Quon et al., 2021).

To ensure safe and effective delivery of auditory stimuli, volume was strictly regulated using a calibrated speaker. Sound levels were maintained within a range of 55 to 60 decibels [dB(A)], adhering to environmental sound standards recommended for neonatal intensive care units. This range was considered sufficient to capture auditory attention without inducing stress or discomfort, as it remained well below the threshold of 70 dB(A) commonly associated with neonatal auditory overstimulation.

Auditory testing was conducted in the routine care environment of the neonatal ward or designated examination room. Measures were taken to reduce background noise and standardize testing conditions. These included positioning the speaker approximately 30 centimeters from the neonate’s head, avoiding sudden loud noises (e.g., alarms, conversations), and excluding data from sessions in which the neonate was crying or visibly agitated.

#### 2.2.5 Evaluation of postnatal neurodevelopment

During follow-up assessments, transcranial ultrasound was repeated to evaluate postnatal neurological development. Specific attention was given to changes in background echogenicity, ventricular morphology, and the development of cerebral sulci and gyri.

#### 2.2.6 Control of physiological confounders

During near-infrared spectroscopy, physiological variables including heart rate, respiratory rate, and body temperature were continuously monitored. Data were excluded if the heart rate exceeded 180 or fell below 100 beats per minute, the respiratory rate exceeded 60 or dropped below 30 breaths per minute, or if body temperature was outside the range of 36.0 °C to 37.5 °C. The TSNIR-3 system sampled at a frequency of 10 Hz, and rSO₂ values were averaged across 5-s intervals to minimize noise. Latency was defined as the time from the onset of auditory stimulation to the first sustained (≥3 s) increase in rSO₂ exceeding 2% of the baseline value.

### 2.3 Statistical analysis

Statistical analyses were performed using SPSS version 22.0 (IBM Corp., Armonk, NY, United States). Spearman’s correlation analysis was employed to evaluate the relationships between gestational age, neonatal birth weight, and corpus callosum growth rate.

The sagittal length and growth rate of the corpus callosum were reported as mean ± standard deviation ( $\bar{x}$ ± s) and compared between groups using the independent samples *t*-test. Categorical variables were summarized as frequencies and percentages, with group differences assessed using the chi-squared test or Fisher’s exact test, as appropriate. A *p*-value ≤0.05 was considered indicative of statistical significance.

Effect sizes were calculated to quantify the magnitude of observed differences. For continuous variables, Cohen’s *d* was used and interpreted according to conventional thresholds (small: 0.2; medium: 0.5; large: 0.8). For categorical variables, odds ratios (OR) with 95% confidence intervals (CI) were reported.

To address the potential confounding effect of unequal distributions of gestational age and birth weight between the hypothyroidism and control groups, an analysis of covariance (ANCOVA) was conducted. Primary outcomes, including corpus callosum length and growth rate between 2 and 6 weeks postnatally, were analyzed using ANCOVA, with group (maternal hypothyroidism vs. control) as the fixed factor and gestational age at birth included as a covariate. This adjustment enabled a comparison of outcomes while statistically controlling for the influence of gestational age, thereby isolating the effect attributable to maternal hypothyroidism status.

## 3 Results

### 3.1 General information

The general clinical characteristics of the study population are summarized in Table 1. A higher proportion of preterm neonates was observed in the hypothyroidism group compared with the control group. Additionally, birth weights were lower among neonates in the hypothyroidism group than in the control group.

**Table 1:** General clinical characteristics of neonates in the hypothyroidism and control groups.

Group	Number of cases *n* (%)	Body weight (g)
Hypothyroidism group	64	
Preterm infants	26 (40.6%)	2000.55 ± 246.54
Full-term infants	38 (59.4%)	2508.02 ± 333.86
Control group	101	
Preterm infants	19 (30.3%)	2113.15 ± 296.27
Full-term infants	82 (69.7%)	2998.02 ± 393.26

Given that gestational age is a key determinant of corpus callosum development, this baseline difference was identified as a potential confounding factor. To isolate the specific effect of maternal hypothyroidism, comparisons of corpus callosum length and growth rate between groups were reanalyzed using ANCOVA, with gestational age at birth included as a covariate.

Following adjustment, the differences in corpus callosum length between the hypothyroidism and control groups remained statistically significant across all time points (*p* < 0.01). Likewise, the difference in corpus callosum growth rate between 2 and 6 weeks of age persisted after adjustment (*p* < 0.001). These findings suggest that maternal hypothyroidism is independently associated with alterations in corpus callosum development, although the possibility of residual confounding cannot be entirely excluded.

### 3.2 Comparative analysis of corpus callosum development

As shown in Table 2, after adjustment for gestational age, neonates in the hypothyroidism group exhibited significantly shorter sagittal corpus callosum lengths than those in the control group at all measured time points (*p* < 0.001 for all). The magnitude of this difference was supported by large effect sizes, indicating strong clinical relevance [at birth: Cohen’s *d* = 1.03, 95% CI (0.71–1.35); at 6 weeks: Cohen’s *d* = 1.27, 95% CI (0.93–1.61)]. Subgroup analysis revealed that this difference was most pronounced among preterm neonates [Cohen’s *d* = 1.42, 95% CI (0.98–1.86)], followed by full-term neonates [Cohen’s *d* = 0.89, 95% CI (0.52–1.26)].

**Table 2:** Sagittal length of the corpus callosum in neonates: comparison between hypothyroidism and control groups (mm/week, $\bar{x}$ ± s).

Group	Birth time					
	0	2	3	4	5	6
Hypothyroidism group						
Preterm infants	32.53 ± 3.15	33.30 ± 2.66	34.62 ± 2.80	35.58 ± 2.68	36.72 ± 2.98	37.88 ± 2.78
Full-term infants	36.83 ± 2.55	37.62 ± 2.82	38.06 ± 2.47	38.91 ± 2.68	39.22 ± 2.65	39.65 ± 2.88
*t*-value	0.38	0.33	0.35	0.38	0.48	0.44
*p*-value	<0.001	<0.001	<0.001	<0.001	<0.001	<0.001
Control group						
Preterm infants	35.20 ± 3.45	35.32 ± 2.86	36.14 ± 2.93	36.88 ± 2.88	37.67 ± 2.90	38.53 ± 2.78
Full-term infants	38.90 ± 2.75	39.71 ± 2.82	40.31 ± 2.87	40.98 ± 2.98	41.87 ± 2.88	42.43 ± 2.88
*t*-value	0.45	0.35	0.38	0.45	0.52	0.48
*p*-value	<0.001	<0.001	<0.001	<0.001	<0.001	<0.001

**Figure 1:**
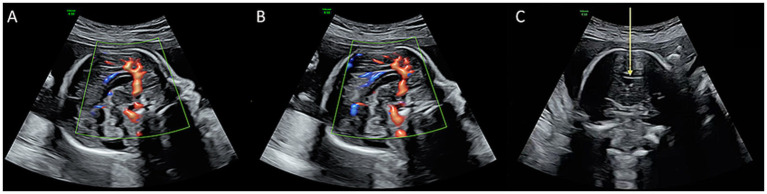
Transcranial ultrasound imaging of the neonatal corpus callosum. **(A)** Echogenicity of the corpus callosum on a normal midsagittal two-dimensional cranial section. **(B)** Color Doppler imaging demonstrating the pericallosal artery (PA) running along the superior margin of the corpus callosum. **(C)** Coronal section showing corpus callosum echogenicity. The arrow indicates the level of the anterior horns of the lateral ventricles on both sides of the frontal lobe. By angling the sound beam approximately 10°, the anterior horns of the lateral ventricles, cavum septum pellucidum (CSP), corpus callosum (seen as a thin, arc-shaped hypoechoic band above the CSP), and the foramen of Monro (interventricular foramen) are visualized. CC, corpus callosum; CSP, cavum septum pellucidum; 3V, third ventricle.

Representative TCUS images illustrating corpus callosum structures are provided in Figure 1.

No statistically significant difference was detected in corpus callosum growth rate between the two groups during the first 0 to 2 weeks after birth (*p* > 0.05). However, from 2 to 6 weeks postnatally, the weekly growth rate of the corpus callosum was significantly lower in the hypothyroidism group compared to the control group (*p* < 0.001). This difference was associated with a large effect size [Cohen’s *d* = 1.51, 95% CI (1.15–1.87)], indicating a substantial developmental disparity during this critical period.

Further analysis demonstrated that corpus callosum sagittal length was positively correlated with both gestational age and birth weight (Table 3).

**Table 3:** Weekly growth rates of the corpus callosum in neonates from birth to 6 weeks: comparison between hypothyroidism and control groups (mm/week, $\bar{x}$ ± s).

Group	Birth time				
	0–2 weeks	2–3 weeks	3–4 weeks	4–5 weeks	5–6 weeks
Hypothyroidism group					
Preterm infants	0.63 ± 0.29	0.68 ± 0.25	34.62 ± 2.80	35.58 ± 2.68	36.72 ± 2.98
Full-term infants	0.87 ± 0.25	0.95 ± 0.25	38.06 ± 2.47	38.91 ± 2.68	39.22 ± 2.65
*t*-value	0.18	1.12	1.28	1.28	1.25
*p*-value	0.72	<0.001	<0.001	<0.001	<0.001
Control group					
Preterm infants	0.83 ± 0.29	0.85 ± 0.24	0.87 ± 0.23	0.88 ± 0.28	0.88 ± 0.20
Full-term infants	1.07 ± 0.25	1.61 ± 0.31	1.56 ± 0.23	1.67 ± 0.26	1.69 ± 0.43
*t*-value	0.26	1.36	1.47	1.38	1.22
*p*-value	0.80	<0.001	<0.001	<0.001	<0.001

Functional assessments also revealed significant group differences. As shown in Table 4, the hypothyroidism group exhibited lower baseline regional cerebral rSO₂ [Cohen’s *d* = 0.97, 95% CI (0.64–1.30)], longer latency in response to auditory stimulation [Cohen’s *d* = 1.12, 95% CI (0.78–1.46)], and reduced peak rSO₂ response values [Cohen’s *d* = 1.05, 95% CI (0.72–1.38)] compared to controls. All differences were statistically significant (*p* < 0.001), with large effect sizes suggesting clinically meaningful alterations in cerebral reactivity.

**Table 4:** Comparison of cerebral tissue oxygen saturation and latency indicators between hypothyroidism and control groups ( $\bar{x}$ ± s).

Group	Oxygen saturation (%)	rSO_2_ response latency (s)	rSO_2_ maximum response value (s)
Hypothyroidism group			
Preterm infants	45.73 ± 8.65	200.50 ± 22.5	3.45. ± 1.64
Full-term infants	69.53 ± 10.0	285.76 ± 26.5	3.99 ± 1.43
Average	57.83 ± 12.9	240.50 ± 16.69	3.70 ± 1.55
*t*-value	0.56	83.85	1.21
*p*-value	<0.001	<0.001	<0.001
Control group			
Preterm infants	64.73 ± 12.3	155.70 ± 11.59	3.83 ± 1.64
Full-term infants	98.53 ± 2.1	175.76 ± 26.02	4.36 ± 1.33
Average	81.63 ± 14.5	165.00 ± 18.79	4.09 ± 1.48
*t*-value	0.56	88.95	1.45
*p*-value	<0.001	<0.001	<0.001

Structural anomalies observed via TCUS are summarized in Table 5. The incidence of abnormal background echogenicity was significantly higher in the hypothyroidism group than in the control group (26.6% vs. 4.0%; *p* < 0.001), corresponding to an OR of 8.69 [95% CI (2.78–27.18)]. Similarly, ventricular broadening was more frequent in the hypothyroidism group (10.9% vs. 2.0%; *p* = 0.016), with an OR of 6.08 [95% CI (1.23–30.11)].

**Table 5:** Incidence of abnormal cranial ultrasound findings detected using TCUS in neonates from hypothyroidism and control groups (*n*).

Group	Control group	Hypothyroidism group	Total
Abnormal background echogenicity			
Present	4 (3 preterm births + 1 full-term)	17 (16 preterm and 1 full-term)	21
Absent	97 (16 preterm births + 81 full-term)	47 (10 preterm + 37 full-term)	144
Ventricle broadening			
Present	2	7	9
Absent	99	57	156
Total	101	64	165

## 4 Discussion

Thyroid hormones are essential endocrine regulators that play a central role in human growth and development. Their synthesis and secretion are controlled through the hypothalamic–pituitary–thyroid axis, a tightly regulated neuroendocrine feedback system. Neurons in the hypothalamus secrete thyrotropin-releasing hormone, which is delivered via the pituitary portal circulation to the anterior pituitary gland. In response, the anterior pituitary releases TSH, which circulates systemically to stimulate thyroid hormone production by the thyroid gland (Mendoza and Hollenberg, 2017).

Although thyroid hormones exert physiological effects across multiple organ systems, their primary function during the perinatal period involves supporting central nervous system development (Mendoza and Hollenberg, 2017). Hormonal deficiency during this time can significantly impair key neurodevelopmental processes such as neuronal differentiation, synaptogenesis, and myelination in the neonatal brain (Tedeschi et al., 2021). During gestation, the fetus relies heavily on maternal thyroid hormones to maintain adequate circulating levels, particularly in the early stages of development.

While the fetal thyroid begins producing hormones around the 12th week of gestation, it does not attain full functional maturity until approximately the 20th week (Landers and Richard, 2017). Prior to this point, maternal thyroid hormones are the primary source of hormonal support for fetal neurodevelopment, particularly in critical brain regions such as the cerebral cortex, hippocampus, and corpus callosum. These hormones contribute to the regulation of neural cell proliferation, migration, and myelination—processes essential for establishing structural and functional integrity in the developing brain.

Existing research has shown that the fetal brain is highly sensitive to fluctuations in maternal thyroid hormone levels. Maternal hypothyroidism has been linked to impaired fetal brain development and may result in measurable structural alterations in the fetal nervous system (Moog et al., 2017).

The corpus callosum is a critical commissural structure that connects the left and right cerebral hemispheres, consisting of approximately 200 to 500 million myelinated and unmyelinated nerve fibers. Anatomically, it is divided into four regions: the rostrum, genu, body, and splenium. Each segment supports distinct interhemispheric functions. The rostrum connects bilateral frontal lobes and is involved in emotional regulation and decision-making processes. The genu links the prefrontal cortices and contributes to higher-order functions such as language and cognition. The body connects the parietal lobes, anterior temporal lobes, and regions of the sensorimotor cortex, facilitating the transmission of somatosensory and motor information. The splenium joins the occipital and posterior temporal lobes and plays a role in visual–spatial integration and associative memory (Richards, 2021).

Given the corpus callosum’s multifaceted involvement in neurocognitive and sensorimotor integration, maternal hypothyroidism during pregnancy may adversely affect its structural and functional maturation. This disruption likely occurs through several interconnected mechanisms driven by inadequate fetal thyroid hormone availability.

Thyroid hormones exert regulatory effects on numerous neurodevelopmental genes through binding to nuclear thyroid hormone receptors (TRα and TRβ). These receptors modulate gene transcription involved in essential processes such as neuronal proliferation, migration, axon guidance, and myelination. During the formation of the corpus callosum, thyroid hormone deficiency reduces the proliferation of neural progenitor cells and disrupts neuronal migration toward the developing callosal region. It also impairs axonal midline crossing by downregulating the expression of key cell adhesion molecules such as L1CAM and interfering with axon guidance signaling pathways, including the Semaphorin and Ephrin families.

Moreover, thyroid hormone deficiency has been shown to downregulate transcription factors vital for oligodendrocyte differentiation, notably Olig2. This impairs the production of major myelin proteins, including myelin basic protein (MBP) and proteolipid protein, leading to delayed or defective myelination of corpus callosum fibers (Lee and Petratos, 2016).

At the molecular level, thyroid hormone modulates the expression of brain-derived neurotrophic factor (BDNF) and nerve growth factor through activation of the cyclic AMP response element-binding protein (CREB) signaling pathway. Disruption of this pathway due to thyroid hormone deficiency may reduce neuronal survival and impair synaptic plasticity, further compromising neurodevelopmental outcomes (Xia et al., 2025). Notably, the regulation of corpus callosum development by thyroid hormone occurs within a defined critical period that spans from the prenatal stage through the first two to three years of postnatal life. While early levothyroxine replacement therapy may mitigate or partially reverse certain structural abnormalities, prolonged or severe deficiency during this window can result in irreversible neurological damage.

Current methods for evaluating neonatal brain structural development primarily include neuroimaging techniques and laboratory screening. Although laboratory tests can reveal functional abnormalities through biochemical markers, their ability to detect specific structural anomalies is limited. As a result, neuroimaging plays a central role in the assessment of fetal and neonatal brain development.

Among available imaging modalities, ultrasound and magnetic resonance imaging (MRI) are most frequently utilized. Fetal MRI offers significant advantages in assessing neonatal brain development, particularly due to its high spatial resolution, which allows for detailed visualization of fine anatomical structures such as gray and white matter, the brainstem, and the cerebellar vermis. Compared to ultrasound, MRI demonstrates superior sensitivity in detecting structural brain abnormalities, including corpus callosum dysgenesis, schizencephaly, and neuronal migration disorders such as lissencephaly. Furthermore, MRI findings are not influenced by maternal factors, enhancing their diagnostic reliability.

However, MRI presents several practical limitations. Scan durations typically range from 30 to 60 min and require the fetus to remain relatively motionless, which may be difficult to achieve without sedation. Additionally, MRI is substantially more expensive—approximately three to five times the cost of ultrasound—and the high cost of MRI equipment limits its availability in primary care settings or rural healthcare facilities. As a result, ultrasound remains a critical and accessible modality for the prenatal and early postnatal evaluation of brain development.

TCUS is a non-invasive imaging technique specifically suited for neonatal assessment, particularly in preterm infants. By utilizing natural acoustic windows such as the anterior fontanelle or thinner cranial bones (e.g., the temporal bone), TCUS enables real-time visualization of intracranial structures through high-frequency ultrasound waves, typically in the range of 7–12 MHz. TCUS is employed to evaluate overall brain development as well as to detect injuries and pathological lesions. Compared with conventional two-dimensional ultrasound, TCUS offers enhanced resolution, improved sensitivity for structural abnormalities, and does not require sedation. Its safety profile and practicality make it especially valuable in routine neonatal care and neurodevelopmental monitoring.

Differences in the sagittal length of the corpus callosum were observed between groups, with more pronounced reductions noted in preterm neonates within the hypothyroidism group. This finding is consistent with known developmental trajectories, as preterm infants exhibit less mature brain structures compared to their full-term counterparts. The corpus callosum undergoes substantial thickening and axonal organization during the mid-to-late stages of gestation; thus, preterm birth results in reduced volume and decreased fiber bundle density. When compounded by insufficient maternal thyroid hormone levels, this underdevelopment is further exacerbated.

In the hypothyroidism group, corpus callosum growth rates did not differ significantly from those of the control group during the first 0 to 2 weeks postnatally (*p* > 0.05). However, from 2 to 6 weeks of age, weekly comparisons revealed statistically significant differences between groups (all *p* < 0.001). Several mechanisms may explain this postnatal divergence. First, the continuation of thyroid dysfunction into the early neonatal period may contribute to suppressed callosal maturation. In cases where neonatal hypothyroidism (e.g., congenital hypothyroidism) is not promptly diagnosed and treated, the resulting T₄ deficiency directly impairs neurodevelopment. Thyroid hormones facilitate synaptogenesis and synaptic pruning via activation of TrkB receptors; their deficiency may disrupt neural network integration and hinder interhemispheric communication mediated by the corpus callosum (Westerholz et al., 2013). Furthermore, thyroid hormone deficiency reduces neonatal metabolic activity, leading to decreased brain glucose utilization and diminished mitochondrial ATP production—both essential for axonal growth and myelination.

Second, the development of the corpus callosum, as the largest white matter tract, is heavily dependent on oligodendrocyte maturation and the formation of myelin. Thyroid hormones are critical in promoting oligodendrocyte differentiation and in stimulating the synthesis of myelin-associated proteins such as MBP. Maternal hypothyroidism may deplete the pool of oligodendrocyte precursors, leading to delayed or suboptimal myelination in the postnatal period. This deficit can manifest as reduced white matter volume or impaired conduction efficiency along callosal fibers (Sheikh, 2020).

Third, thyroid hormone deficiency can interfere with molecular signaling pathways and gene expression programs essential for brain development. Through nuclear thyroid hormone receptors, these hormones regulate the transcription of key neurodevelopmental genes, including *FOXP2* and *PAX8*. Prolonged intrauterine deficiency may lead to persistent suppression of genes responsible for myelination via epigenetic mechanisms such as DNA methylation. In addition, T₄ supports neuronal survival and axonal outgrowth by promoting BDNF secretion. In hypothyroid conditions, downregulation of the BDNF–TrkB signaling pathway may impair the extension of corpus callosum fiber tracts (Marty et al., 2022).

Findings in the control group supported these associations, as a positive correlation was observed between corpus callosum sagittal length, gestational age, and birth weight. This is consistent with previous studies reporting that neonates with maternal hypothyroidism tend to show reduced size in anterior and central callosal regions, while the posterior corpus and splenium regions may appear relatively preserved or enlarged—a pattern aligned with the present study’s conclusions (Willoughby et al., 2014).

In this study, corpus callosum length was measured as the distance from the genu to the splenium in the midsagittal plane. In addition to structural findings, the assessment of neonatal cerebral reactivity revealed lower rSO₂, prolonged latency, and reduced maximal rSO₂ response in the hypothyroidism group. These observations are consistent with prior research linking prenatal thyroid hormone insufficiency to altered cerebral oxygenation and impaired hemodynamic responses. However, the interpretation of near-infrared spectroscopy data warrants caution, as cerebral rSO₂ is modulated by multiple physiological factors—including cerebral blood flow, systemic oxygenation, and oxygen consumption—that could not be fully accounted for in this study. Furthermore, the use of a single auditory stimulus (Mozart’s *Sonata for Two Pianos*, K. 448) may limit the generalizability of reactivity assessments, as neonatal responses can vary depending on stimulus type, duration, and intensity.

TCUS identified a higher incidence of abnormal brain findings in the hypothyroidism group compared with controls, which may be partially attributable to the greater proportion of preterm infants in this group. Preterm neonates typically exhibit less mature brain morphology, which may present as increased parenchymal echogenicity, lateral ventricular enlargement, and less pronounced sulcal and gyral patterns—features that are readily detectable by TCUS. Given its accessibility, non-invasiveness, and high sensitivity, TCUS holds significant clinical utility for the early detection of atypical brain development.

Several limitations of this study must be acknowledged. The control group consisted of neonates admitted for conditions such as pneumonia, hyperbilirubinemia, or respiratory distress. These conditions may independently affect cerebral oxygenation, perfusion, and early neurodevelopment, potentially introducing bias in the assessment of corpus callosum growth and cerebral reactivity. Although rigorous inclusion and exclusion criteria were applied, and relevant clinical variables were statistically adjusted for, the potential influence of these conditions cannot be entirely excluded.

Another limitation is the absence of subgroup analyses based on the characteristics of maternal hypothyroidism. Previous studies have reported that overt hypothyroidism, early gestational onset, and inadequate treatment are associated with increased neurodevelopmental risks (Moog et al., 2017). In the present cohort, 43.8% of mothers had overt hypothyroidism and 35.9% did not receive treatment during pregnancy. However, the study design did not allow for separate evaluation of their individual contributions to neonatal corpus callosum development. Future investigations should incorporate stratified analyses based on hypothyroidism severity, timing of diagnosis, and treatment adequacy to better elucidate potential dose–response relationships between maternal thyroid dysfunction and offspring neurodevelopment.

It is also important to consider that some of the observed differences in brain development between groups may be attributable to the higher prevalence of preterm birth and lower birth weight in the hypothyroidism group. While adjustments for gestational age and birth weight were performed, maternal hypothyroidism remained significantly associated with atypical corpus callosum development, suggesting an independent effect. Nonetheless, residual confounding cannot be fully ruled out. Potential unmeasured variables, such as maternal socioeconomic status, dietary iodine intake, tobacco or alcohol use during pregnancy, concurrent medications, and other metabolic conditions including gestational diabetes, may have influenced both maternal thyroid function and fetal neurodevelopment, contributing to the associations observed in this study.

## 5 Conclusion

Maternal hypothyroidism during pregnancy is associated with reduced corpus callosum length, slower postnatal growth rate, and altered cerebral reactivity in neonates. These findings suggest that maternal thyroid dysfunction may adversely influence early structural and functional brain development. However, given the observational nature of the study, the results indicate an association rather than a definitive causal relationship. Residual confounding, such as unmeasured maternal metabolic conditions, environmental exposures, or lifestyle factors, may partly account for the observed differences and should be considered when interpreting these outcomes.

## Data Availability

The original contributions presented in the study are included in the article/supplementary material, further inquiries can be directed to the corresponding author.
